# Selenium’s Role in Neuroprotection Against Stroke-Induced Inflammation

**DOI:** 10.7759/cureus.52205

**Published:** 2024-01-13

**Authors:** Ernesto Navarro Garcia, Sebastian Leon, Nilo Alvarez Toledo

**Affiliations:** 1 Nanoscience Technology Center, University of Central Florida, Orlando, USA; 2 Department of Physiology/Neuroscience, St. George's University, St. George's, GRD; 3 Burnett School of Biomedical Sciences, University of Central Florida, Orlando, USA; 4 Department of Physiology/Neuroscience, St. George's University, St. George’s, GRD

**Keywords:** cell studies, cytotoxicity, viability, reactive oxygen species, neuro inflammation, selenium preventive effect, selenium functions

## Abstract

Acute ischemic stroke (AIS) incidence across the globe is on the rise, and the deleterious effects have not yet been improved with the use of current pharmaceuticals. Tissue plasminogen activator (tPA) has many risks and time constraints, making it difficult to use even as the standard treatment. Selenium deficiency and stroke incidence have a strong linear correlation among various populations. Using the ADME-Tox software, selenious acid absorption in brain cells, tissue, and interstitium was modeled under ischemic conditions to determine the bioavailability of selenium (Se) in the brain using various IV (intravenous) infusion doses. Additionally, we studied the cytotoxicity of selenious acid and selenourea on human dermal fibroblasts (HDF) and lung carcinoma cells (A549) to determine the overall growth and toxicity of different body cell lines to account for systemic side effects of IV infusion. Our data suggest that selenium can reach a dose-dependent concentration of 1.5µg/L or 250µg/L in brain cells within two hours of a one-time IV infusion, showing the ability to reach brain vasculature. Furthermore, cell viability can be maintained between 95% and 100% using 1nM and 0.5nM concentrations of selenious acid.

## Introduction

Stroke, a prevalent life-threatening condition globally, prompts concern. In 2019, the American Heart Association estimated hypertension's prevalence at 46% among Americans. Each day, we witness an average of 389.4 stroke-related deaths, with an anticipated rise to 23 million incidents by 2030 [1]. This surge necessitates improved therapeutic strategies focused on improved recovery rates and decreased neuronal volume loss. Presently, tPA is among the favored drugs to treat ischemic attacks, yet debates persist regarding heightened bleeding risks and contraindications [2-4].

Selenium's physiological role has shown promise in stroke animal models, acting at a transcriptional level. Its neuroprotective properties trigger a broad transcriptional response involving TFAP2 (transcription factor AP-2) and Sp1 (specific protein 1), boosting glutathione peroxidase 4 (GPX4) synthesis. Selenium binds to DNA promoters, inducing GPX4 transcription and countering oxidative damage from ischemia [5]. Studies on selenium serum levels and dietary intake further support its neuroprotective effects. A collection of selenium serum levels and dietary intake studies further correlate the neuroprotective effects of selenium.

Research by Yuanyuan Shi et al. highlighted selenium's role via transcriptional genes. HCA and hemin are both capable of inducing excitotoxicity and neuronal damage, respectively. However, these were effectively countered by sodium selenite (SeO3-2) due to selenium's impact on selenoprotein genes, notably GPX4. This emphasized GPX4's crucial role, as selenium alone couldn't protect neurons without GPX4’s presence. Notably, selenium didn't shield against RSL3, a direct GPX4 expression inhibitor [6]. Additionally, neurons with silenced GPX4 expression succumbed to ferroptosis when exposed to HCA or hemin, yet co-treatment with selenium safeguarded them. This safeguard is due to selenium's ability to reduce free iron and hydroxyl radicals via Fenton reactions.

In a mouse model with induced ICH, intracerebroventricular selenium injection showcased reduced cell death and functional recovery after seven days, attributed to selenium's role in curbing ferroptosis. Selenium's potential to reduce free iron and subsequent hydroxyl radicals via Fenton chemistry further bolsters its protective capacity.

Although these results show promise, using mouse models poses a scientific challenge due to the vast differences between humans and mice. The administration of selenium still poses a challenge due to the invasive nature of this research. A new approach to delivery must be investigated. In this work, we present the effects of selenium based compounds in lung cells and dermal fibroblasts to further elucidate the potential systemic related side effects of selenious acid and selenourea if given via IV infusion. 

## Materials and methods

Current literature review

The reviews for selenium, stroke correlation, and current research on the potential use of selenium as a pharmaceutical were done using a total of five databases: PubMed, Google Scholar, Scopus, Embase, and NIH. These databases were also searched for case-control studies and cohort studies on serum levels of selenium and ischemic stroke outcomes published up to August 30, 2022. Key search words included “stroke statistics,” “stroke,” “selenium,” “stroke and selenium,” “case studies,” “cohort studies,” "stroke and selenium medications," and “selenium pharmaceuticals.” The papers were chosen based on their relevance to the aim of this paper, which is solely on cerebrovascular disease. These papers focus on natural levels of selenium in serum, dietary levels, and their correlation with stroke incidence. Papers including other systemic diseases and other comorbidities were excluded to maintain this study related to cerebrovascular-specific events. These exclusion criteria were applied to minimize co-existing comorbidities and underlying health factors that may not be related to selenium.

ADME-Tox simulation

The ADME-Tox software was acquired from Thermo Fisher Scientific. The software modeled the amount of selenium intake by brain cells, brain tissue, and interstitium. A stroke model was mimicked by the absorption, distribution, metabolism, and excretion (ADME) program with specific ion concentrations of sodium, potassium, and iron. The modeled patient was standardized to 70kg. The absorption of selenium was measured across brain tissue, brain cells, and brain interstitium over a period of 24 hours via a one-time IV infusion of selenious acid.

Reagents

Selenious acid (98%, CAS: 7783-00-8, MW: 128.97 g/mol) and the A549 cell line were purchased from Sigma-Aldrich (30500 Spruce Street, St. Loui, MO 63103, USA). All other reagents, such as selenourea (99%, CAS: 630-10-4, MW: 123.02), were purchased from Thermo Fisher Scientific (30 Bond Street, Ward Hill, MA 01835, USA).

Synthesis and characterization

Selenious acid and Selenourea were weighed and dissolved in deionized water, forming 1 mg/mL, respectively. These solutions were then diluted accordingly for cell culture studies in DMEM, supplemented with 1% antibiotics and 10% fetal bovine serum (Sigma-Aldrich). A Tecan M200 Pro plate reader was used for analyzing Resazurin-based cytotoxicity assays.

Optical microscopy studies

Cells were treated with either selenious acid or selenourea, juxtaposed with a non-treated group of cells and a set of cells treated with dimethyl sulfoxide. After 24 hours of exposure to the treatments, the media was changed, and the cells were imaged at 10x magnification. The cells were then treated with resazurin, incubated for two hours, and read for fluorescence. A Nikon Eclipse Ts2 Inverted Light Microscope was used for capturing cell images at 10x.

Cytotoxicity studies

A cytotoxicity assay was performed with resazurin on human dermal fibroblasts (HDF) and A549 cell lines at various concentrations based on clinically recommended doses for selenium supplementation. We used 0.07mg/kg and 12µg/kg of two selenium compounds. These doses vary from the recommended 60µg/day [7] to determine the lowest and maximum doses at which the trace metal can reach the brain’s milieu. The resazurin Alamar blue reagent was used to assess the metabolic output of cells that were exposed to selenious acid and selenourea at various concentrations. A549 lung carcinoma cells and HDF cells were seeded at 1x104 cells per well on two 96-well plates, given various concentrations of selenious acid formulations, and then assessed using the Alamar blue assay.

## Results

Collective data gathered in Table 1 shows a clear inverse correlation between serum levels of selenium and stroke incidence. Studies in populations from India, China, Canada, and the USA have shown the significance of normal serum levels of selenium as a neuroprotective agent. Overall, stroke incidence among all populations was lower if adequate serum levels of selenium were present.

**Table 1:** Serum selenium levels and stroke-related events across various populations.

Author	Year	Journal	Cases	Findings
Wang Z et al. [8]	2022	The American Journal of Clinical Nutrition	n=20,702	Inverse relation between selenium level and stroke incidence.
Ramezani M et al. [9]	2021	Neurologist	n=50	Selenase functions as an antioxidant and improves neurological deficits.
Mirończuk A et al. [10]	2021	Nutrients	n=210	Lower ratios of Cu/Se have worse outcomes in acute ischemic attacks.
Hu XF et al. [11]	2019	Journal of American Heart Association	n=12,095	High blood selenium concentrations are related to low stroke incidence.
Hu XF et al. [12]	2017	Journal of Trace Elements in Medicine and Biology	n=2077	Reverse association between stroke prevalence and serum/dietary selenium.
Hu H et al. [13]	2021	BMC	n=1255	Inverse correlation between selenium levels and first stroke incidence.

Furthermore, in-vitro and in-vivo studies shown in Table 2 demonstrate the effectiveness of selenium nanoparticle therapy for neuronal preservation in cell and mouse models. However, these nanoparticles currently have several drawbacks dictated by the FDA due to unknown long-term effects of aggregation, possible toxicities, and expensive synthetic materials.

**Table 2:** The latest selenium-based studies on neuroprotection and mechanisms of action.

Author	Year	Journal	Findings
Alim I et al. [6]	2022	Springer: Neurochemical Research	Selenium can alleviate cerebral damage from I/R injuries.
Zhang S et al. [14]	2011	Journal of Nutritional Biochemistry	Neuronal cells treated with selenium derivatives protect them against ROS.
Amani H et al. [15]	2019	Scientific Reports 9	Biodegradable nanoparticles increase neuronal survival in in-vivo models.

**Figure 1:**
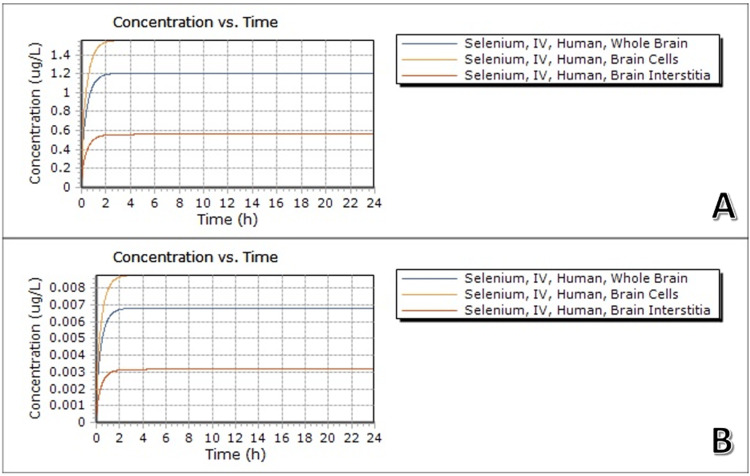
Selenium absorption by brain tissue, cells, and interstitium was obtained via ADME software on a standardized 70kg patient. (A) Selenium was administered via IV 1x per 24-hour period. The dose of selenium was 0.000070 grams, or 0.07mg/k, or 5000µg/day (B) Selenium was administered by IV 1x per 24-hour period at a dosage of 0.0003948mg/kg, or 27µg/day.

To further contribute to the existing data on selenium-based compounds, our data via ADME-Tox, shown in Figure 1, suggests that selenious acid can easily penetrate brain tissue, brain cells, and brain interstitium at minuscule doses within the first two hours of an IV infusion. At a higher dose of 5000µg/day, the absorption is reached at almost the same time as at a lower dose of 27µg/day, with varying dose-dependent concentrations achieved. These absorption rates suggest that achieving therapeutical conditions at a guided dosage of 60mcg/day [7] is possible without complex-carrying vessels.

**Figure 2:**
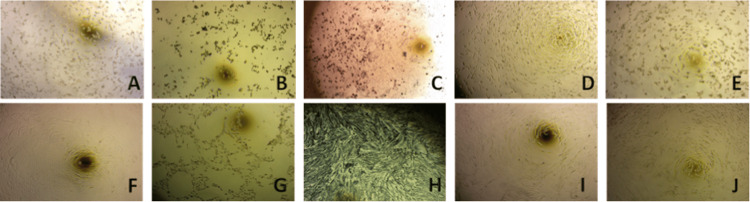
Cellular responses to selenium in acidic (selenious acid) and basic (selenourea) formulations, all images were obtained via light microscopy at x10 resolution. (A) A549 cell as a growth control. (B) A549 cells exposed to selenious acid at 1000nM. (C) A549 cells exposed to selenourea at 1000nM. (D) A5F49 cells exposed to selenious acid at 0.5nM. (E) A549 cells exposed to selenourea at 0.5nM. (F) HDF cell as a growth control. (G) HDF cells exposed to selenious acid at 1000nM. (H) HDF cells exposed to selenourea at 1000nM. (I) HDF cells exposed to selenious acid at 0.5nM. (J) HDF cells exposed to selenourea at 0.5nM.

**Figure 3:**
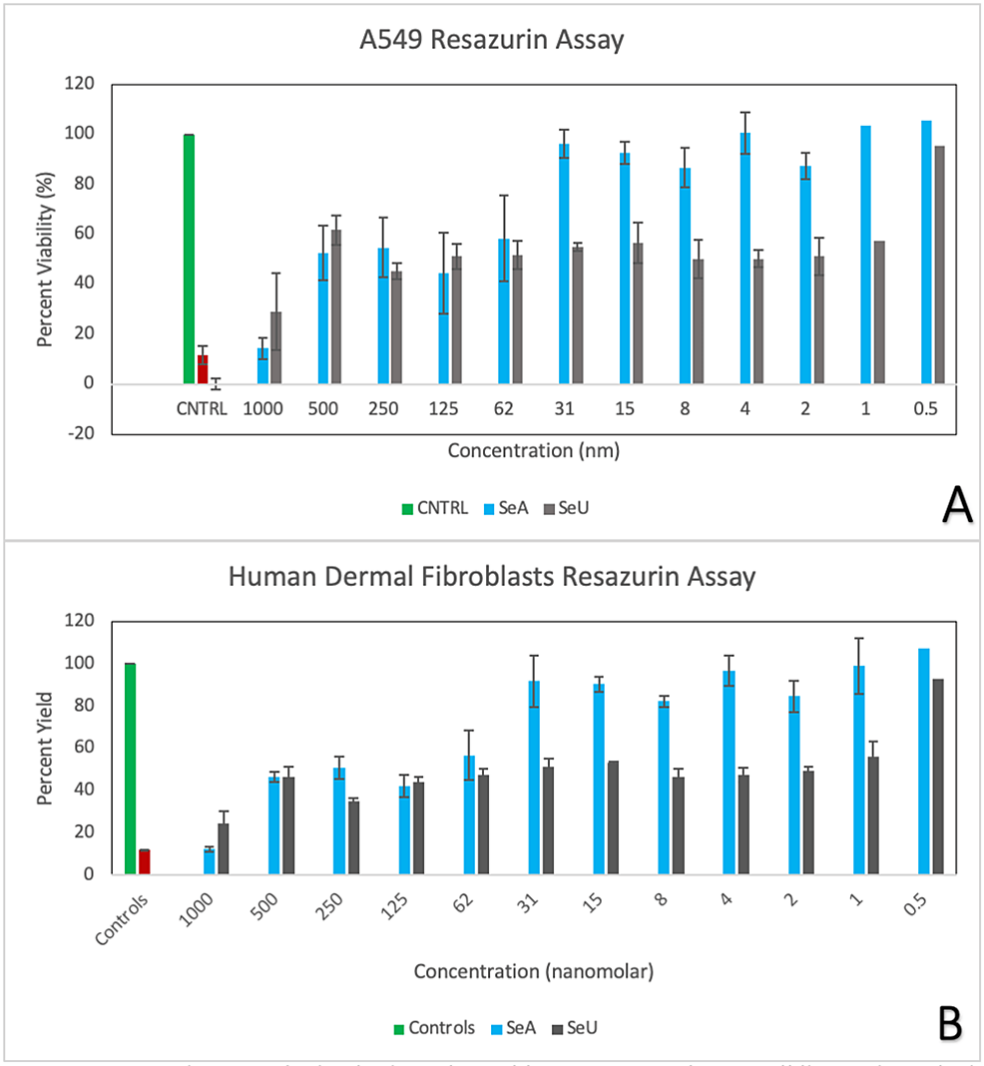
A cytotoxic assay was obtained using Alamar blue on A549 and HDF cell lines using selenious acid and selenourea. (A) A549 cellular viability after exposure to selenourea and selenious acid at various nanomolar concentrations. (B) HDF cellular viability after exposure to selenourea and selenious acid at various nanomolar concentrations.

Furthermore, our toxicity assays in HDF and A549 cell lines show that selenious acid can preserve and even induce cellular growth at low nanomolar concentrations. These results are of particular importance as they highlight the effects of selenious acid once it’s in systemic bloodstream and other organ tissues. Light microscopy Figures 2D, 2I show cellular proliferation at 0.5nM of selenious acid. These same cellular responses are shown in Figure 3A. In contrast, selenourea appears toxic at all nanomolar concentrations, as shown in Figures 2C, 2E, 2H, 2J, 3B.

The distinction between the two chemical states of selenium is essential due to the different metabolic states that exist through various illnesses. In the setting of ischemic stroke, the pH environment ranges from 6.0 to 6.9, with a slight alkalotic increase in a chronic setting and a fast regression to an acidic state over time [16-18]. The metabolic state induced by ischemia favors the selenious acid formulation as it will help degrade it and act on cells. Once selenium interacts with cells, it may work on cell preservation via protein translation of antioxidative properties.

## Discussion

Selenium, a trace metal crucial for various bodily functions [19], is notably recognized for its established role as an antioxidant. Recent observations have spotlighted its involvement in hypothalamic function [20]. The amalgamation of this research and extensive literature evaluations yields pivotal insights into selenium's role in acute ischemic stroke and inflammation attributed to reactive oxygen species (ROS). Notably, establishing a correlation between stroke incidence and serum selenium levels across diverse populations afflicted by stroke was a primary pursuit [8].

Compelling literature strongly suggests an inverse relationship between adequate to elevated selenium levels and stroke incidence. This association was discerned across varied populations in India, China, Canada, and the USA, encompassing a substantial sample size of 36,389 individuals [9-13]. These findings demonstrate selenium's potential neuroprotective role and its plausible contribution to stroke prevention.

Expanding on this promising avenue, selenium-based nano-carriers have been synthesized to investigate their impact on cellular processes, employing both in-vitro and in-vivo stroke-induced models. Compounds derived from selenious acid exhibit the capacity to induce protein translation, generating ROS scavengers [6]. Nano-encapsulation forms enable these compounds to effectively penetrate cells [15], preserving neuronal density post-stroke induction in murine models [15].

Further substantiating selenium's utility, our research employing ADME-Tox methodologies demonstrates the ability of selenium infusion via intravenous administration to permeate neuronal cells, interstitium, and brain parenchyma at both high and low doses (Figures 1A, 1B). However, the concentration attained exhibits a dose-dependent pattern. Notably, this study indicates that the FDA-approved dose of 60mcg/day [7] should effectively permeate and reach various components within the brain.

Moreover, our cytotoxicity assays using A549 and HDF cell lines reveal differential effects of selenious acid and selenourea. Selenious acid maintains cellular bioavailability between 90% and 100% at low nanomolar concentrations (Figures 2D, 2I, 3A), while selenourea proves toxic across all nanomolar concentrations (Figure 3B). In an ischemia-induced acidic brain environment [16-18], selenious acid demonstrates efficacy through degradation, allowing molecular-level action [6]. Our studies demonstrate the safety of selenious acid on non-neuronal cell lines, as evidenced by observations on HDF and A549 cells. This assessment carries significance when contemplating potential adverse effects on other organ systems once selenium is present in systemic circulation. 

Furthermore, the utilization of intravenous selenious acid infusion mitigates concerns related to long-term side effects arising from nanoparticle aggregation, legal standards, and metabolic processes [21,22]. Our ongoing investigations continue to emphasize the need to consider selenium-based compounds or appropriate intravenous selenium infusions as adjunct therapeutic strategies for acute ischemic strokes or analogous brain conditions sharing similar pathophysiology.

Limitations

Certain limitations are to be noted in our study. The ADME-Tox software uses a computerized theoretical model, which is vastly different from a real biological simulation. Likewise, the software uses a standardized patient model weighing 70 kg, which could affect the volume of distribution of selenium and its concentration rates. In the clinic, patients' weights vary per case, and the necessary dose adjustment should be considered. However, based on the simulation, it is safe to say that because selenium can permeate at very low and high doses, the necessary adjustment can be made. Lastly, the IV infusion is for selenium, not the acidic or basic forms, both of which have been shown to behave differently in our study. 

## Conclusions

The collective findings from selenium studies strongly indicate the pivotal role played by selenium-based compounds in safeguarding neurological health. These conclusions draw from an array of sources, encompassing human serum studies as well as ongoing investigations delving into more effective methods of delivering selenium to the brain using mouse models. Our research, alongside existing literature, confidently asserts that selenium exhibits notable ease of absorption by brain tissues, regardless of whether it's administered at high or low doses via IV infusion. Notably, the rate of absorption doesn’t undergo significant fluctuations when comparing high and low doses. However, the concentrations acquired do vary, contingent upon the dosage. As the IV dose of selenious acid increases, so does its serum concentration in the bloodstream. Selenium absorption culminates in peak tissue, parenchymal, and neuronal concentrations within 2 hours, maintaining a plateau thereafter.

The presence of selenium-based compounds in systemic circulation showcases the safety profile of selenious acid for both HDF and A549 cell lines. Our investigations highlight that even at low nanomolar concentrations, selenious acid poses no threat to dermal and lung tissues. Moreover, our findings emphasize that at low doses, the cell viability of these lines can be upheld between 95% and 100%, underscoring the relative safety of this compound within systemic circulation. Further exploration into the cytotoxic properties of selenious acid and selenoruea as they enter systemic circulation necessitates comprehensive studies on hepatic and renal cells. Collectively, the current body of literature, combined with our research, not only underscores the potential use of selenium as a primary adjunct in treating cerebral inflammation but also emphasizes the imperative need for in-depth human studies to elucidate its full potential.

## References

[1] Benjamin EJ, Muntner P, Alonso A (2019). Heart disease and stroke statistics-2019 update: a report from the American Heart Association. Circulation.

[2] Toyoda K, Koga M, Iguchi Y (2019). Guidelines for the intravenous application of recombinant tissue-type plasminogen activator (alteplase), the second edition, October 2012: a guideline from the Japan Stroke Society. Neurol Med Chir (Tokyo).

[3] Lees KR, Bluhmki E, von Kummer R (2010). Time to treatment with intravenous alteplase and outcome in stroke: an updated pooled analysis of ECASS, ATLANTIS, NINDS, and EPITHET trials. Lancet.

[4] Hacke W, Donnan G, Fieschi C (2004). Association of outcome with early stroke treatment: pooled analysis of ATLANTIS, ECASS, and NINDS rt-PA stroke trials. Lancet.

[5] Shi Y, Han L, Zhang X, Xie L, Pan P, Chen F (2022). Selenium alleviates cerebral ischemia/reperfusion injury by regulating oxidative stress, mitochondrial fusion and ferroptosis. Neurochem Res.

[6] Alim I, Caulfield JT, Chen Y (2019). Selenium drives a transcriptional adaptive program to block ferroptosis and treat stroke. Cell.

[7] Mayo Foundation for Medical Education and Research (2023). Selenium supplement (oral route) proper use. Mayo Clinic.

[8] Wang Z, Ma H, Song Y (2022). Plasma selenium and the risk of first stroke in adults with hypertension: a secondary analysis of the China Stroke Primary Prevention Trial. Am J Clin Nutr.

[9] Ramezani M, Simani L, Abedi S, Pakdaman H (2021). Is selenium supplementation beneficial in acute ischemic stroke?. Neurologist.

[10] Mirończuk A, Kapica-Topczewska K, Socha K, Soroczyńska J, Jamiołkowski J, Kułakowska A, Kochanowicz J (2021). Selenium, copper, zinc concentrations and Cu/Zn, Cu/Se molar ratios in the serum of patients with acute ischemic stroke in Northeastern Poland-a new insight into stroke pathophysiology. Nutrients.

[11] Hu XF, Stranges S, Chan LH (2019). Circulating selenium concentration is inversely associated with the prevalence of stroke: results from the Canadian Health Measures Survey and the National Health and Nutrition Examination Survey. J Am Heart Assoc.

[12] Hu XF, Sharin T, Chan HM (2017). Dietary and blood selenium are inversely associated with the prevalence of stroke among Inuit in Canada. J Trace Elem Med Biol.

[13] Hu H, Bi C, Lin T (2021). Sex difference in the association between plasma selenium and first stroke: a community-based nested case-control study. Biol Sex Differ.

[14] Zhang S, Luo Y, Zeng H, Wang Q, Tian F, Song J, Cheng WH (2011). Encapsulation of selenium in chitosan nanoparticles improves selenium availability and protects cells from selenium-induced DNA damage response. J Nutr Biochem.

[15] Amani H, Habibey R, Shokri F, Hajmiresmail SJ, Akhavan O, Mashaghi A, Pazoki-Toroudi H (2019). Selenium nanoparticles for targeted stroke therapy through modulation of inflammatory and metabolic signaling. Sci Rep.

[16] Kobatake K, Sako K, Izawa M, Yamamoto YL, Hakim AM (1984). Autoradiographic determination of brain pH following middle cerebral artery occlusion in the rat. Stroke.

[17] Back T., Hoehn M., Mies G. (2000). Penumbral tissue alkalosis in focal cerebral ischemia: Relationship to energy metabolism, blood flow, and steady potential. Ann. Neurol.

[18] Pignataro G, Simon RP, Xiong ZG (2007). Prolonged activation of ASIC1a and the time window for neuroprotection in cerebral ischaemia. Brain.

[19] Roman M, Jitaru P, Barbante C (2014). Selenium biochemistry and its role for human health. Metallomics.

[20] Toh P, Nicholson JL, Vetter AM, Berry MJ, Torres DJ (2022). Selenium in bodily homeostasis: hypothalamus, hormones, and highways of communication. Int J Mol Sci.

[21] Jan Simak (2022). Investigation of potential toxic effects of engineered nanoparticles and biologic microparticles in blood and their biomarker applications. US Foo Dru Admin.

[22] Gupta R, Xie H (2018). Nanoparticles in daily life: applications, toxicity and regulations. J Environ Pathol Toxicol Oncol.

